# Quality of Life of Short-Statured Children Born Small for Gestational Age or Idiopathic Growth Hormone Deficiency Within 1 Year of Growth Hormone Treatment

**DOI:** 10.3389/fped.2019.00164

**Published:** 2019-04-29

**Authors:** Julia Quitmann, Janika Bloemeke, Neuza Silva, Monika Bullinger, Stefanie Witt, Ilker Akkurt, Desiree Dunstheimer, Christian Vogel, Volker Böttcher, Ursula Kuhnle Krahl, Markus Bettendorf, Eckhard Schönau, Susanne Fricke-Otto, Alexandra Keller, Klaus Mohnike, Helmuth-Günther Dörr

**Affiliations:** ^1^Center for Psychosocial Medicine, Institute for Medical Psychology, University Medical Center Hamburg-Eppendorf, Hamburg, Germany; ^2^Faculty of Psychology and Education Sciences, Center for Research in Neuropsychology and Cognitive Behavioral Intervention, University of Coimbra, Coimbra, Portugal; ^3^Children and Adolescent Endocrinology, MVZ am AKK GmbH, Hamburg, Germany; ^4^Clinic for Children and Adolescents, Augsburg Hospital, Augsburg, Germany; ^5^Clinic for Children and Adolescent Medicine, Chemnitz Hospital, Chemnitz, Germany; ^6^Endocrinology Clinic, Frankfurt am Main, Germany; ^7^Diabetes Center, Gauting, Germany; ^8^Center for Children and Adolescent Medicine, University Clinic of Heidelberg, Heidelberg, Germany; ^9^Pediatric Endocrinology, University Clinic of Cologne, Cologne, Germany; ^10^Center for Children and Adolescent Medicine, HELIOS Hospital, Krefeld, Germany; ^11^Kinderzentrum am Johannisplatz, Leipzig, Germany; ^12^University Children's Clinic, Otto von Geuricke University, Magdeburg, Germany; ^13^Clinic for Children and Adolescents, Erlangen-Nürnberg Universtiy, Erlangen, Germany

**Keywords:** short stature, health-related quality of life, growth hormone treatment, ISS, IGHD, QoLISSY

## Abstract

Aside from clinical endpoints like height gain, health-related quality of life has also become an important outcome indicator in the medical field. However, the data on short stature and health-related quality of life is inconsistent. Therefore, we examined changes in health-related quality of life in German children with idiopathic growth hormone deficiency or children born small for gestational age before and after 12 months of human growth hormone treatment. Children with idiopathic short stature without treatment served as a comparison group. At baseline, health-related quality of life data of 154 patients with idiopathic growth hormone deficiency (*n* = 65), born small for gestational age (*n* = 58), and idiopathic short stature (*n* = 31) and one parent each was collected. Of these, 130 completed health-related quality of life assessments after 1-year of human growth hormone treatment. Outcome measures included the Quality of Life in Short Stature Youth questionnaire, as well as clinical and sociodemographic data. Our results showed that the physical, social, and emotional health-related quality of life of children treated with human growth hormone significantly increased, while untreated patients with idiopathic short stature reported a decrease in these domains. Along with this, a statistically significant increase in height in the treated group can be observed, while the slight increase in the untreated group was not significant. In conclusion, the results showed that human growth hormone treatment may have a positive effect not only on height but also in improving patient-reported health-related quality of life of children with idiopathic growth hormone deficiency and children born small for gestational age.

## Introduction

Short stature is defined as having a height that is lower than two standard deviation scores below the mean height for age and gender of the reference population and about 3% of the children in a population are of small stature (1). It is a common symptom for a variety of conditions—some of which can be of endocrine nature such as idiopathic growth hormone deficiency (IGHD). Although growth hormone deficiency may have congenital or acquired causes, IGHD may be diagnosed if such deficiency of growth hormone is found without any apparent cause resulting in a short statured body (2). However, in most cases, the pathological cause of short stature in children and adolescents cannot be determined, which is described as idiopathic short stature (ISS) (3). Individuals diagnosed with ISS are born normal sized, but have a low growth velocity and often have a family history of short stature (4). If infants have a birth weight and/or length of <2 standard deviation scores they are diagnosed with small for gestational age (SGA) (3). Without catch-up growth in the first 2 years these children remain short (5).

Short stature is reported to cause various deficiencies in physical aspects of daily life and in emotional- and social well-being (2, 6). Furthermore, chronic psychosocial stress, stigmatization and social isolation, frequent bullying and less social competencies than in children who are of normal height have been reported in short statured children impacting on the children's health-related quality of life (HRQOL) (7–9). However, the data on short stature and HRQOL is inconsistent. Some studies reported deficits in HRQOL in short statured children diagnosed with IGHD or SGA (6, 10, 11), others reported no significant differences from the reference population (12).

The therapeutic options for pediatric endocrinologists to treat short children with human growth hormone (hGH) are limited to a few indications (13–15). Among them are children with IGHD and SGA, whereas the treatment of short children with ISS without apparent pathology is not approved by the European Medicines Agency. The positive effects of hGH treatment such as inducing catch-up growth and achieving adequate adult height within the normal range for mid-parental target height have been shown in children with IGHD in many studies (16–20). The hGH treatment of children with SGA leads to a gain in adult height of 7–10 cm and also improves body composition (21–23).

Aside from clinical endpoints like height gain, HRQOL has also become an important outcome indicator in the medical field (24). The concept of HRQOL is described as the subjective perception of health including physical, social and emotional aspects of well-being (25). Although the use of hGH treatment in short statured children and adolescents has been shown to increase height, it is still not clear if this treatment positively affects the HRQOL of this population. Some studies report an indirect relationship between height gain and HRQOL in children with IGHD or SGA and ISS who received hGH treatment (14, 26, 27), while other studies report inconsistent findings in differences of HRQOL after hGH treatment (28). Patients with IDGH or ISS undergoing hGH treatment showed no differences in HRQOL compared to norm populations (29). Also Theunissen et al. (12) found no improvement in HRQOL between treated and untreated children with ISS. Furthermore, Visser-van Balen et al. (30) showed that hGH treatment did not improve psychosocial functioning in a small sample of young adults with ISS or born with SGA compared to an untreated control group. It is important to consider, that these studies used a mix of various generic and/or short-stature specific instruments to assess HRQOL and studies were designed differently with regard to patient samples and control, which makes the interpretation of results quite challenging. However, the Quality of Life for Short Statured Youth (QoLISSY) questionnaire is a disease-specific instrument, designed to assess HRQOL in children and adolescents with IGHD, SGA or ISS from the patients' and parents' perspective and offers the opportunity to adequately assess HRQOL in this patient population (31).

Because of the inconsistency in study results and methodology concerning HRQOL improvement due to hGH treatment in short statured children, we raise the research question: Does 1-year hGH treatment improves HRQOL in children with IGHD or SGA from the patients' and parents' perspective? Therefore, we conducted a prospective multicenter observational study in Germany using the disease-specific QoLISSY instrument to assess the HRQOL of short children with IGHD or SGA before and 1 year after the start of hGH treatment, taking gender differences into account. Since hGH treatment of short children with ISS is not approved in Germany, these untreated children served as a comparison group. Although reports on psychosocial outcomes due to hGH treatment are inconsistent, we hypothesized that HRQOL significantly improves in the intervention group (children with IGHD or SGA), while no significant HRQOL changes were expected in the comparison group (children with ISS).

## Materials and Methods

### Subjects and Procedures

Initially, all German children diagnosed with IGHD or SGA where hGH treatment was intended were planned to be included in this observational study. Thus, all children hospitals and pediatric endocrinologist across Germany were informed about the study. However, due to lacking endorsement of the clinicians, only 11 pediatric endocrinologists from 9 children hospitals and 2 medical practices in Germany agreed to recruit patients for the study. Between 2013 and 2016 the patients were consecutively recruited at initial visits at a pediatric endocrinologist. The participating clinicians were instructed to inform all newly admitted patients diagnosed with IGHD, SGA and ISS aged 8–18 years about the study. With a power of 80% to detect changes in the QoLISSY *Total score* (sum of the scales *Physical, Social*, and *Emotional*) and considering dropouts, the desired sample should include each *n* = 160 children/adolescents in the intervention and comparison group and *n* = 240 parents. If patients agreed to participate in the study, a written informed consent was obtained from all patients and their parents/legal guardians. The diagnoses of IGHD or SGA were made according to current guidelines (32). The patients in the intervention group were treatment-naïve and treated with an approved brand of hGH in Germany. Since children with ISS could not be treated with hGH in Germany, short children with the diagnosis of ISS without hGH served as the comparison group. Patients with any other diagnosis that result in short stature (e.g., Turner syndrome) and/or displayed a lack of adequate linguistic competency were not included in the study.

Children (aged 8–18) and one parent each, as well as parents of younger children (aged 4–7) from the intervention group, were asked to complete the pen and paper short stature specific QoLISSY questionnaire (31) before the start of hGH treatment at baseline and 1-year after the start of treatment during the waiting period on-site at the respective clinic or the endocrinologist. The comparison group of untreated children/adolescents with ISS also answered the QoLISSY questionnaire at two measurement points with an equivalent time interval.

In addition to the report on HRQOL as assessed with the QoLISSY, self-reported socio-demographic data included patients' and parents' sex, date of birth and nationality. Physician-reported clinical data included diagnosis (ISS, IGHD or SGA), treatment status and height at time of diagnosis and at time of assessment.

Since this study was a multicenter study, the study was approved by the respective ethics committees of the centers, namely by the Hamburg Medical Chamber, the Saxon medical chamber, the Hessen medical chamber, the Nordrhein medical chamber and by the ethics committee of the Friedrich-Alexander University Erlangen–Nürnberg, of the University of cologne, of the University Hospital Magdeburg and of the Ludwig-Maximiliams-University Munich. All subjects gave written informed consent in accordance with the Declaration of Helsinki.

### Measures

The QoLISSY questionnaire was developed according to standardized guidelines, including: (a) focus-groups with item generation; (b) pilot-test with cognitive debriefing; and (c) field test with retest (33). The questionnaire for children is applicable from the age of eight and older, while the parent-report version is available for parents of children aged 4–18 years. The children's version (total of 50 items) consists of three core domains (*Physical, Emotion*, and *Social* HRQOL) as well as three additional domains (*Coping, Beliefs*, and *Treatment*). The parents' version (total of 66 items) is used to obtain observer reports on identical domains and assesses additional parent-specific domains (*Future* and *Effects on parents*). Mean QoLISSY raw scores were computed for each scale when 80% of the data were available (i.e., missing data lower than 20% of the values for each scale) and subsequently transformed into a score between 0 and 100, with higher values representing higher HRQOL (31). The QoLISSY *Total score* was calculated as the mean of the three core domain scores (*Physical, Social*, and *Emotional*). Psychometric performance of the QoLISSY questionnaire within this prospective trial is satisfactory, as recently reported by Bloemeke et al. (34).

### Statistical Analysis

The statistical analyses were performed with the Statistical Package for the Social Sciences, v.21 (IBM Corp., Armonk, NY), with the significance level settled at *p* < 0.05. All assessed data of the participants was pseudonymized for analysis. Therefore, the name and other identification characteristics were replaced by an indication to prevent the identification of the study participant.

Descriptive statistics for socio-demographic and clinical variables (i.e., mean and SD for continuous variables; frequencies and percentages for categorical variables) were calculated. Auxological data (i.e., height) was calculated also in standard deviation scores (SDS). Reference data for height SDS were taken from Kromeyer-Hausschild et al. (35) who used the LMS formula by Cole (36) for calculation. The homogeneity of sample characteristics across the diagnostic groups at baseline was examined by independent-samples analysis of variance (continuous variables) or χ^2^ tests (categorical variables).

To examine changes in the primary outcome (HRQOL) within the course of hGH treatment, a repeated measures MANCOVA for the QoLISSY scales and a repeated measures ANCOVA for the QoLISSY *Total score* were conducted. Measurement points (i.e., baseline vs. 1-year follow-up) were entered as within-subjects factors, while the treatment status (intervention vs. comparison group) was entered as between-subjects factor. The children's age and time difference between both measurement points were entered into the models as covariates. Furthermore, a two-factorial repeated measurement ANCOVA for the QoLISSY *Total score* was conducted. Measurement points (i.e., baseline vs. 1-year follow-up) were entered as the within-subjects factors. Diagnoses (IGHD, SGA, ISS) and gender, were entered as between-subjects factors. Children's age at baseline and time difference between both measurement points were also entered in the models as covariates. Pillai's trace was used as the test statistic for the MANCOVA/ANCOVA calculations, with increasing positive values suggesting effects that contribute more to the model (37). Effect-size measures were presented for the comparison analyses, with $\eta_{\text{p}}^{2}$ ≥ 0.01, $\eta_{\text{p}}^{2}$ ≥ 0.06, and $\eta_{\text{p}}^{2}$ ≥ 0.14 considered as small, medium, and large effects, respectively (38).

## Results

### Sample Characteristics

**Figure 1:**
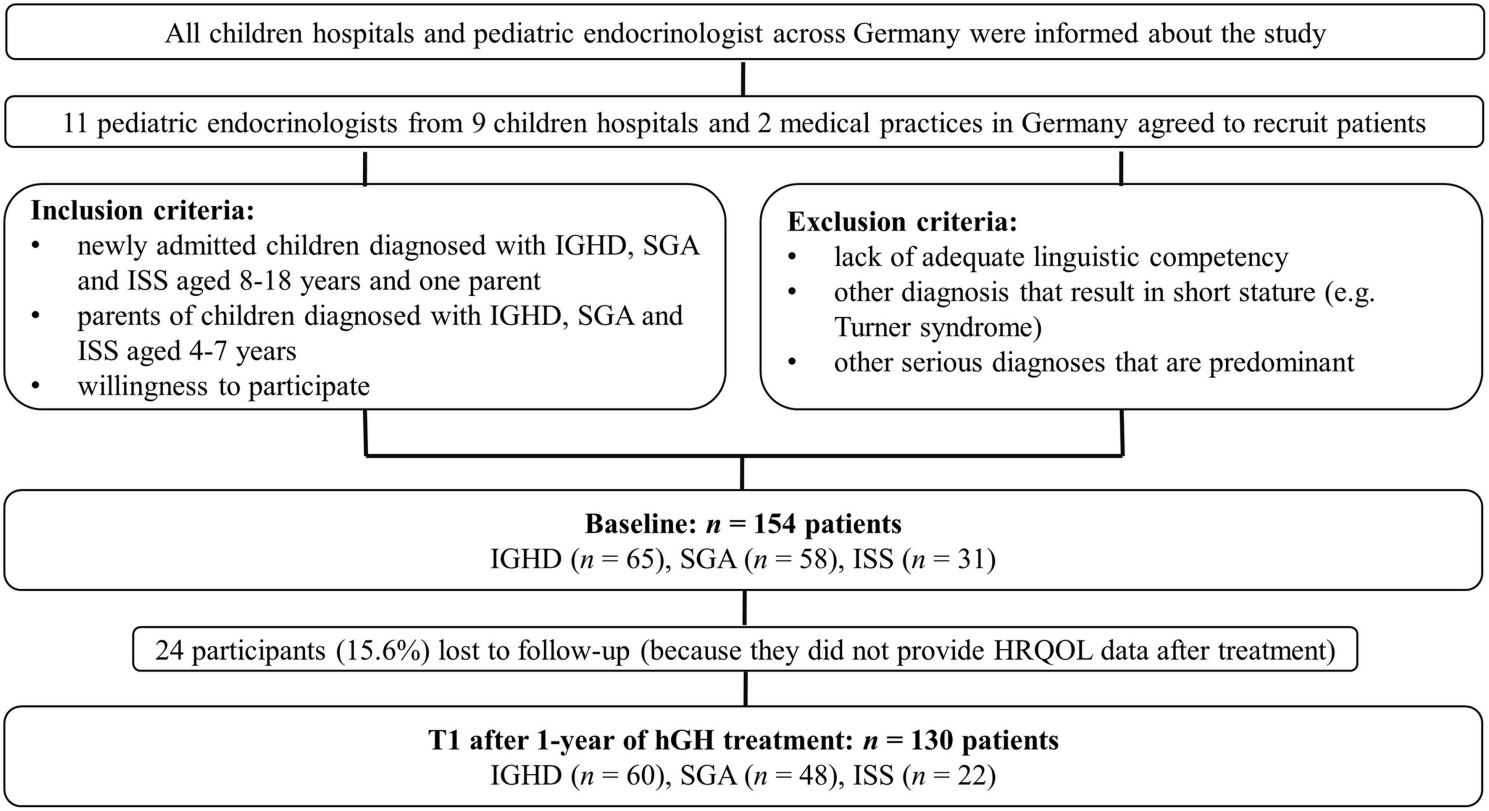
Flowchart of participant selection.

At baseline, 154 patients with diagnosed IGHD (*n* = 65), SGA (*n* = 58), or ISS (*n* = 31) were assessed (66 QoLISSY patient-reports from children/adolescents between 8 and 18 years of age and 152 QoLISSY proxy-reports from parents of 4–18 year-old children). Of these, 130 participants, diagnosed with IGHD (*n* = 60), SGA (*n* = 48), or ISS (*n* = 22), also completed the QoLISSY questionnaire after 1-year of hGH treatment (70 patient-reports and 126 parent-reports at 1-year follow-up), with 24 participants (15.6%) lost to follow-up because they did not provide HRQOL data after treatment. Figure 1 provides an overview about the participant selection. The sociodemographic and clinical characteristics of participants at baseline and 1-year follow-up are presented in Table 1. The distribution of children's sex across the diagnostic groups was homogeneous at baseline, $\chi_{\left(2\right)}^{2}$ = 5.93, *p* = 0.06. However, differences were found on children's age, *F*_(2)_ = 9.21, *p* < 0.01, with SGA patients being significantly younger than children/adolescents with IGHD or ISS.

**Table 1:** Sample characteristics and QoLISSY *Total scores* at baseline and 1-year after human growth hormone treatment.

		**Baseline (*****n*** **= 154)**			**1-year follow-up (*****n*** **= 130)**		
		**IGHD (*n* = 65)**	**SGA (*n* = 58)**	**ISS (*n* = 31)**	**IGHD (*n* = 60)**	**SGA (*n* = 48)**	**ISS (*n* = 22)**
Sex, *n* (%)	Male	48 (73.8%)	33 (56.9%)	16 (51.6%)	44 (73.3%)	29 (60.4%)	11 (50.0%)
	Female	17 (26.2%)	25 (43.1%)	15 (48.4%)	16 (26.7%)	19 (39.6%)	11 (50.0%)
Chronological Age (years), M (SD)		8.09 (3.34)	6.55 (2.64)	9.45 (3.49)	9.22 (3.37)	7.81 (2.76)	10.68 (3.24)
	Missing	–	–	–	1 (1.7%)	–	–
Age group, *n* (%)	4–7 years	34 (52.3%)	41 (70.7%)	7 (22.6%)	26 (43.3%)	28 (58.3%)	4 (18.2%)
	8–12 years	22 (33.8%)	15 (25.9%)	16 (51.6%)	22 (36.7%)	18 (37.5%)	10 (45.5%)
	13–17 years	9 (13.8%)	2 (3.4%)	8 (25.8%)	11 (18.3%)	2 (4.2%)	8 (36.4%)
	Missing	–	–	–	1 (1.7%)	–	–
Height (cm), M (SD)		117.11 (17.44)^a^	108.01 (13.45)^a^	126.24 (18.86)^a^	127.04 (17.53)^c^	118.89 (13.65)^c^	132.48 (17.77)^c^
	Missing	–	1 (1.7%)	1 (3.2%)	2 (3.3%)	2 (4.2%)	1 (4.5%)
Height deviation (SDS), M (SD)		−2.61 (0.61)^b^	−2.65 (0.63)^b^	−2.11 (0.51)^b^	−1.91 (0.64)^d^	−2.05 (0.67)^d^	−2.06 (0.73)^d^
	Missing	–	1 (1.7%)	1 (3.2%)	3 (5.0%)	2 (4.2%)	1 (4.5%)
Treatment length (months), M (SD)		–	–	–	12.64 (1.93)	12.25 (2.43)	12.73 (2.76)
	Missing	–	–	–	1 (1.7%)	–	–
QoLISSY Total score^e^, M (SD)	Child report	*n* = 26	*n* = 15	*n* = 23	*n* = 33	*n* = 18	*n* = 18
		49.48 (27.2)	45.64 (23.3)	64.86 (20.0)	58.90 (24.9)	68.10 (16.6)	60.87 (24.2)
	Parent report	*n* = 62	*n* = 54	*n* = 31	*n* = 57	*n* = 46	*n* = 22
		51.10 (26.3)	48.49 (19.8)	60.20 (23.4)	56.38 (24.6)	58.63 (21.7)	60.62 (25.1)

*IGHD, idiopathic growth hormone deficiency; SGA, small for gestational age; ISS, idiopathic short stature; SDS, Standard deviation score; M, Mean; SD, standard deviation*.

a *Independent-samples analysis of variance: Significant difference between the diagnosis groups in height at T0 (F_(2)_ = 12.74, p ≤ 0.01)*.

b *Independent-samples analysis of variance: Significant difference between the diagnosis groups in height SDS at T0 (F_(2)_ = 8.70, p ≤ 0.01)*.

c *Independent-samples analysis of variance: Significant difference between the diagnosis groups in height at T1 (F_(2)_ = 5.59, p ≤ 0.01)*.

d *Independent-samples analysis of variance: No significant difference between the diagnosis groups in height SDS at T1 (F_(2)_ = 7.21, p = 0.48)*.

e *Sum of the scales Physical, Social, Emotional*.

Regarding clinical characteristics, a significant difference between height SDS across the diagnosis groups was found at baseline, with significant differences between IGHD (SDS = −2.61) and ISS (SDS = −2.11) as well as between SGA (SDS = −2.65) and ISS (SDS = −2.11), with patients with ISS having a statistically higher SDS reaching nearer to a normal height. Regardless of the treatment status, all patients grew during the study period. Within the diagnosis groups IGHD and SGA, there was a significant increase in height SDS and height (*p* ≤ 0.01) from baseline to 1-year follow-up. Height gain in height SDS after 1-year of treatment was *M* = 0.70 (*SD* = 0.45) in patients with IGHD, *M* = 0.58 (*SD* = 0.43) in patients with SGA and *M* = 0.09 (*SD* = 0.49) in ISS (Table 1).

### HRQOL Before the Start of hGH Treatment

At baseline, *t*-tests revealed significant differences in the QoLISSY *Total score* between the children in the intervention group (children to be treated) and the comparison group (children that will remain untreated). Children in the intervention group rated their HRQOL significantly lower (*M* = 51.23, *SD* = 25.68) before treatment start, than the comparison children who remained untreated (*M* = 64.86, *SD* = 20.00), *t*_(−2.23)_ = 68, *p* ≤ 0.05. Also in the parent-reports, parents of children in the intervention group rated the *Total QoL* significantly lower (*M* = 50.31, *SD* = 23.82) before treatment start, than parents of children in the comparison group (*M* = 61.72, *SD* = 23.06), *t*_(−2.49)_ = 160, *p* ≤ 0.05.

### HRQOL Between Treated and Untreated Children Within the Course of hGH Treatment

#### Patient-Reports

**Figure 2:**
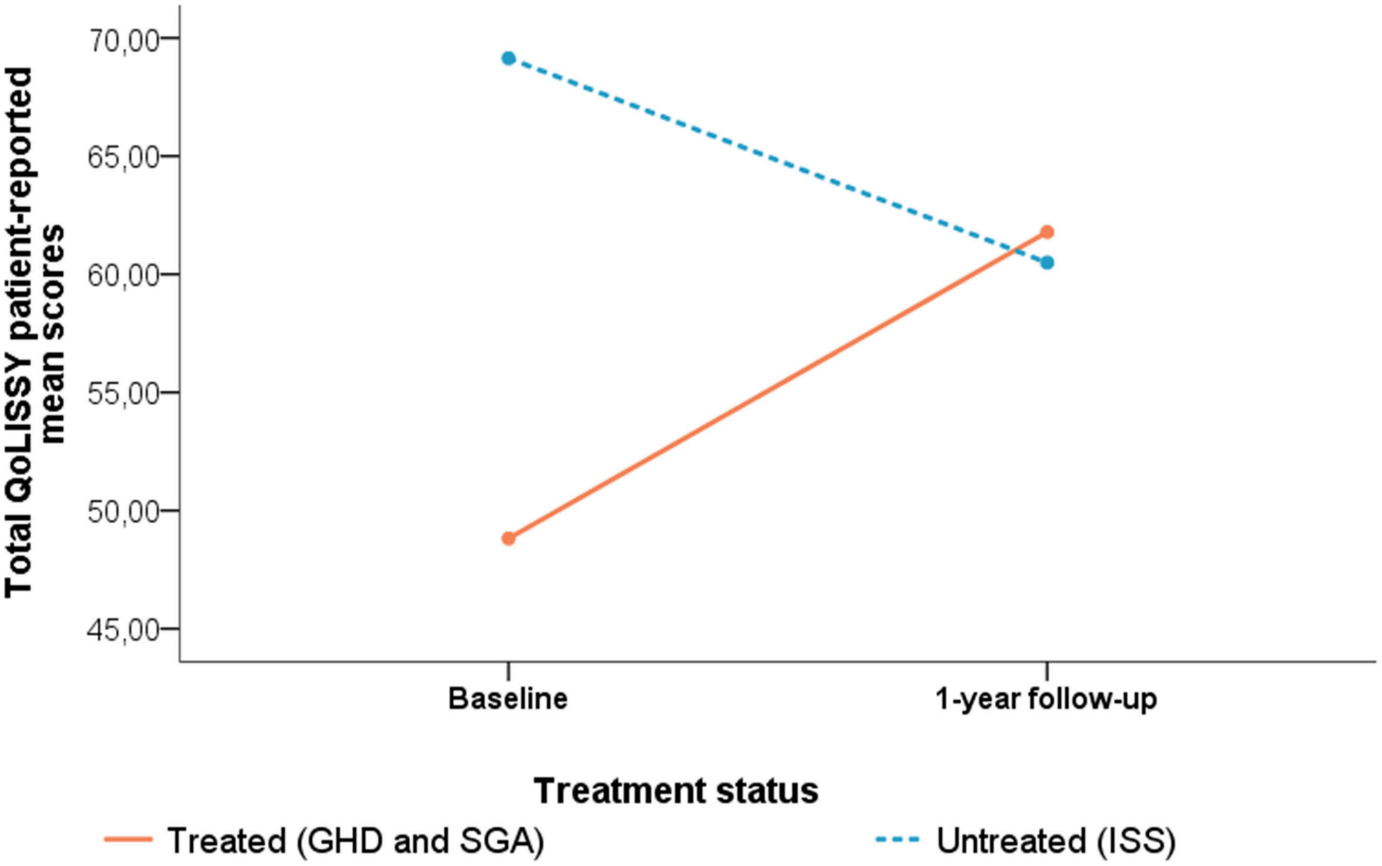
Changes in patient-reported QoLISSY *Total score* from baseline to 1-year after start of hGH treatment for the intervention group (patients with IGHD and SGA) and comparison group (untreated patients with ISS). Two-factorial repeated measurement ANCOVA for the QoLISSY *Total score*. Covariates appearing in the model were evaluated at the following values: Age at baseline = 10.91 years; Time difference between baseline and 1-year follow-up = 12.74 months.

Over the duration of hGH treatment, the repeated measures MANCOVA for the patient-reported QoLISSY scales revealed a multivariate significant interaction effect between time and treatment status, Pillai's trace = 0.27, *F*_(5, 43)_ = 3.24, *p* = 0.01, $\eta_{\text{p}}^{2}$ = 0.27 (Table 2). Subsequent univariate analyses (Table 3) showed that children with IGHD or SGA who were treated with hGH reported a significant increase in the *physical, social*, and *emotional* HRQOL domain from baseline to 1-year after start of the treatment, while untreated patients with ISS reported a decrease in *physical, social*, and *emotional* HRQOL over time. These results were corroborated by the univariate ANCOVA for repeated measures for the QoLISSY *Total score*, with a significant interaction effect between time and treatment status, *F*_(1, 50)_ = 9.72, *p* < 0.01, $\eta_{\text{p}}^{2}$ = 0.16. This trend held true even after controlling for the effects of age at baseline, *F*_(1, 50)_ = 0.03, *p* = 0.86, $\eta_{\text{p}}^{2}$ = 0.00, and treatment length, *F*_(1, 50)_ = 3.97, *p* = 0.05, $\eta_{\text{p}}^{2}$ = 0.07 (Figure 2).

**Table 2:** Multivariate effects of the repeated measures MANCOVA and effects of the multifactorial repeated measurement ANCOVA.

**Report**	**Variables**	**Pillai's trace**	***F***	**df**	**Error df**	**Sig. (*p*-value)**	**$\eta_{\text{p}}^{2}$**
Patient-report	Time^a^	0.17	1.83	5	43	0.12	1.76
	Treatment status^a^	0.13	1.33	5	43	0.26	1.34
	Time^*^ treatment status^a^	0.27	3.24	5	43	0.01	0.27
	Time^*^ diagnose^*^ gender^b^	0.14	3.78	2	46	0.03	0.14
	Time^*^ diagnose^*^ age groups^b^	0.01	0.18	2	46	0.83	0.008
Parent-report	Time^a^	0.19	2.91	7	88	0.01	0.14
	Treatment status^a^	0.14	2.07	7	88	0.06	0.14
	Time^*^ treatment status^a^	0.06	0.85	7	88	0.55	0.06
	Time^*^ diagnose^*^ gender^b^	0.03	1.81	2	114	0.16	0.03
	Time^*^ diagnose^*^ age groups^b^	0.06	1.92	4	111	0.11	0.06

a *Repeated measures MANCOVA for the QoLISSY scales with children's age and time difference between both measurement points as covariates*.

b *Two-factorial repeated measurement ANCOVA for the QoLISSY Total score with children's age at baseline and time difference between both measurement points as covariates*.

**Table 3:** Univariate analyses of covariance of HRQOL changes from baseline and 1-year follow-up between treated (idiopathic growth hormone deficiency and small for gestational age) and untreated (idiopathic short stature) patients.

		**Treated with hGH**	**Untreated**			
		**Mean (SD)**	**Mean (SD)**	***F***	***p***	**$\eta_{\text{p}}^{2}$**
**Patient-reports**						
Physical	T0	48.99 (26.96)	69.91 (18.22)	8.51	<0.01	0.15
	T1	62.65 (22.85)	61.94 (27.57)			
Social	T0	45.67 (26.64)	66.42 (20.42)	14.76	<0.01	0.24
	T1	62.04 (25.86)	58.51 (23.70)			
Emotional	T0	47.06 (24.86)	70.71 (26.56)	11.68	<0.01	0.20
	T1	61.24 (23.29)	62.18 (26.80)			
Coping	T0	60.98 (22.29)	67.78 (21.62)	2.44	0.13	0.05
	T1	60.56 (16.19)	57.08 (18.03)			
Beliefs	T0	41.86 (30.03)	68.06 (31.21)	3.23	0.08	0.06
	T1	46.59 (33.88)	56.60 (28.72)			
Total score	T0	48.88 (24.17)	69.01 (19.50)	9.72	<0.01	0.16
	T1	61.60 (22.88)	60.88 (24.20)			
**Parents-reports**						
Physical	T0	46.78 (25.54)	62.50 (25.90)	0.14	0.71	0.00
	T1	54.11 (25.04)	63.92 (26.11)			
Social	T0	48.20 (24.64)	58.62 (24.46)	0.79	0.38	0.01
	T1	55.49 (25.73)	58.50 (27.39)			
Emotional	T0	47.88 (24.37)	58.19 (23.58)	0.85	0.36	0.01
	T1	55.89 (24.63)	60.16 (27.56)			
Coping	T0	48.30 (17.03)	59.89 (21.49)	1.56	0.22	0.02
	T1	50.42 (15.68)	55.18 (24.01)			
Beliefs	T0	51.92 (28.97)	61.25 (32.23)	0.24	0.62	0.00
	T1	50.88 (29.08)	57.19 (36.01)			
Future	T0	52.05 (30.38)	70.25 (25.31)	3.33	0.07	0.03
	T1	59.73 (28.37)	63.56 (29.28)			
Effects on parents	T0	49.48 (22.10)	59.84 (24.41)	0.25	0.62	0.00
	T1	55.62 (21.70)	58.99 (27.36)			
Total score	T0	50.56 (23.57)	60.97 (22.46)	1.33	0.25	0.01
	T1	57.34 (23.31)	60.63 (25.05)			

Repeated measures MANCOVA for the QoLISSY scales and a repeated measures ANCOVA for the QoLISSY Total score. Children's age and time difference between both measurement points were entered into the models as covariates

*Repeated measurement: T0, baseline; T1, 1-year follow-up; IGHD, idiopathic growth hormone deficiency; SGA, small for gestational age; ISS, idiopathic short stature; SD, standard deviation; F, f -value; p, p-value; $\eta_{\text{p}}^{2}$, partial eta square*.

#### Parent-Reports

Regarding the parent-reports, there was a significant multivariate effect of time, Pillai's trace = 0.19, *F*_(7, 88)_ = 2.91, *p* = 0.01, $\eta_{\text{p}}^{2}$ = 0.19. The univariate analyses for the main effect of time revealed a significant improvement from baseline to 1-year after start of treatment on the *Effects on parents* scale, for both intervention and comparison groups, *F*_(1, 94)_ = 7.75, *p* < 0.01, $\eta_{\text{p}}^{2}$ = 0.08.

No multivariate effects of treatment status, Pillai's trace = 0.14, *F*_(7, 88)_ = 2.07, *p* = 0.06, $\eta_{\text{p}}^{2}$ = 0.14, or interaction effects between time and treatment status, Pillai's trace = 0.06, *F*_(7, 88)_ = 0.85, *p* = 0.55, $\eta_{\text{p}}^{2}$ = 0.06, were found (Table 2). The univariate analyses for the interaction effect are presented in Table 3.

**Figure 3:**
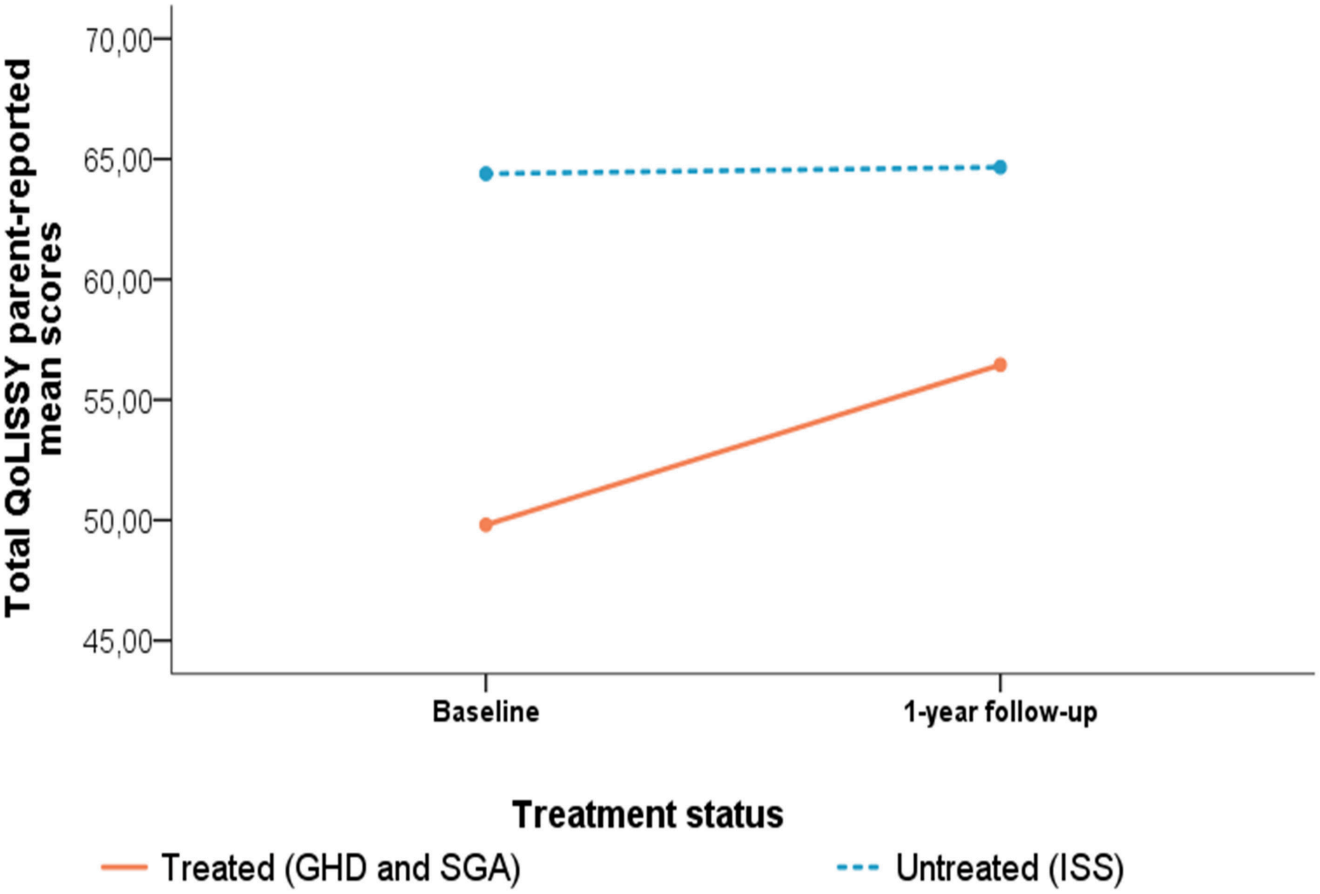
Changes in parent-reported QoLISSY *Total score* from baseline to 1-year after start of hGH treatment for the intervention group (patients with IGHD and SGA) and comparison group (untreated patients with ISS). Two-factorial repeated measurement ANCOVA for the QoLISSY *Total score*. Covariates appearing in the model were evaluated at the following values: age at baseline = 7.81 years; Time difference between baseline and 1-year follow-up = 12.45 months.

The ANCOVA for the parent-reported QoLISSY *Total score* (Figure 3) showed no significant main effects of time, *F*_(1, 118)_ = 1.02, *p* = 0.31, $\eta_{\text{p}}^{2}$ = 0.01. Interaction effects between time and treatment status were also not found when controlling for age at baseline and treatment length, *F*_(1, 118)_ = 1.33, *p* = 0.25, $\eta_{\text{p}}^{2}$ = 0.01. However, a significant main effect of treatment status was found for the parent-reported QoLISSY *Total score, F*_(1, 118)_ = 5.83, *p* = 0.02, $\eta_{\text{p}}^{2}$ = 0.05, with parents of treated children reporting lower HRQOL than parents of untreated children, independently of time of assessment.

### HRQOL Between Diagnoses and Gender Within the Course of hGH Treatment

#### Patient-Reports

Looking at the total HRQOL differences between diagnoses and gender, a multifactorial repeated measurement ANCOVA with age and the time difference between baseline and 1-year after treatment as covariates revealed a significant main effect for the interaction between time, diagnose and gender, Pillai's trace = 0.14, *F*_(2, 46)_ = 3.78, *p* < 0.05, $\eta_{\text{p}}^{2}$ = 0.14. Girls with IGHD and SGA reported an increase in their HRQOL, which was particularly high in girls born with SGA, while girls with ISS reported a decrease in their HRQOL. Boys reported a slight increase in their HRQOL across all diagnosis groups.

#### Parent-Reports

In the parent-reports, these results were not statistically significant (Table 2). Regarding HRQOL differences between the age groups (4–7 years, 8–12 years, >13 years) over time, there were no significant interaction effects between time, age groups and diagnose in patient-reports, nor in the parent-reports (Table 2).

## Discussion

Results of the present study showed that improved growth of short statured children with IGHD and SGA who were treated with hGH for 1 year positively affects their short-term HRQOL compared to untreated short statured children diagnosed with ISS.

Already at baseline, children with IGHD and SGA who were about to start treatment were significantly shorter than patients with ISS. Further, these patients show a statistically significant lower HRQOL before treatment than children with ISS. From the children's point of view, our findings showed a significant interaction effect between time and treatment status. Children who were treated with hGH, reported a significant increase in physical, social, and emotional HRQOL from baseline to 1-year after treatment, while untreated patients reported a decrease in physical, social, and emotional HRQOL over time. The total HRQOL differences between the diagnoses and the gender revealed a significant interaction effect between time, diagnosis and gender. Treated girls with IGHD or SGA reported an increase in their HRQOL, while girls diagnosed with ISS from the comparison group, reported a decrease in their HRQOL. In boys, a slight increase in HRQOL across all diagnosis groups was observed. Hence, based on the results of this study, the positive influence of hGH treatment on growth (39–41) can now be supplemented by an increase in HRQOL in this specific patient group, considering the lack of potential confounders such as comorbidities, socioeconomic background or social inclusion, which were not assessed within the study. The finding of a reduction in HRQOL in the untreated comparison group was unexpected. A possible explanation for this might be that these children and adolescents are aware of treatment options for short statured people, such as hGH treatment, but are unable to get access to this kind of treatment due to their diagnosis which is not approved for hGH treatment in Germany. Considering the fact that these children also contacted pediatric endocrinologists and seeking for treatment, this might negatively impact on their HRQOL over time.

Analyzing the parents data, there was also a significant multivariate main effect of time, but no multivariate main effects of treatment status or interaction effects between time and treatment status, were found. Still, it seems important to us that the univariate analyses for the main effect of time revealed a significant improvement from baseline to 1-year after start of treatment on the *Effects on parents* scale, for both intervention and comparison groups. This is an important finding since the results from another large European cross-cultural study allowed us the determination of caregiving stress as an important risk factor for parent's impaired quality of life, as well as its mediating role between children's psychosocial functioning and parents' outcomes (42). Another study by Brod et al. (43) about treatment burden described parents not only as substantially impacted by worry surrounding their children's treatment administration, but also by their worry about causing the child pain and medication costs. This study confirms parental feelings of sadness about the need for treatment, guilt, and frustration with injection administration. Taking into account these findings and the results of this and our former studies, we agree with the recommendations by Gardner et al. (44), who suggest that clinicians must consider factors beyond height when selecting and preparing patients and parents for treatment. In accordance, we emphasize the importance of a shared decision-making paradigm in which clinicians, patients, and their parents make decisions for and against treatment with hGH, based on the best available evidence with full knowledge about evolving scientific controversies.

### Limitations and Strength

To our knowledge, this is the first study to examine the effects of hGH treatment on HRQOL in a longitudinal study design within a multicenter study in Germany using a short stature specific HRQOL questionnaire. Nevertheless, our study has several limitations that should be acknowledged. When interpreting the results, it should be considered that the sample size between the groups was uneven. Furthermore, the sample is very selective and represents a homogeneous group of patients and parents that is not necessarily representative of the overall target population, since all of them contacted growth clinics and hence recognized that short stature may be a problem, that treatment options are available, and that treatment may be necessary. Moreover, the selected age groups of this study need to be critically discussed. Usually, it is uncommon that adolescents aged >13 years old are treated with hGH because treatment is more effective at a prepubertal stage and children that are short-statured are typically diagnosed in early childhood (45). However, in this study, adolescents aged >13 years were also included in the sample as patients with a late diagnosis. In light of this limitation, it needs to be considered that only a developmental delay could be present. Still, this limitation was unavoidable due to small sample sizes of the study, and therefore this age group was also included in the analysis.

Besides, the desired treatment duration of 1 year was difficult to adhere to because the participating families did not show up exactly 1 year after treatment started in the participating center where they received the treatment. Hence, the time window from assessing the HRQOL at baseline to 1-year after start of treatment differed across participants and was subsequently controlled in the analyses. Although 1 year of hGH treatment might be too short to assess long-term effects, this time period is long enough to detect differences in HRQOL. This effect was also presented in a recent study by González Briceño et al. (46), who assessed HRQOL with the QoLISSY questionnaire before and after 1 year of growth hormone treatment in French children ≥4 years.

Another important limitation is that due to organizational issues, we were not able to include a normal stature, age-matched control group of children or a group of untreated children with IGHD or SGA for comparison. Our study was, however, conducted in a hospital/clinical practice setting and hence provides real world insight into the effects of hGH treatment on HRQOL in children with IGHD and SGA. Furthermore, some participants, especially patients diagnosed with ISS, which served as a comparison group, were already near normal height (see Table 1). Besides, comparing children who received treatment to children who received no treatment due to drug regulation might also evoke frustration in children (i.e., children with ISS) who are short, but are not able to receive treatment. This might explain, why children with ISS report a decrease in their HRQOL.

Nevertheless, our study included a larger number of children compared with previous studies that have examined the effects of hGH treatment on the quality of life in children with IGHD and SGA (27, 47, 48). In addition, our study used the cross-culturally validated short stature specific QoLISSY questionnaire, which takes into consideration both the child's and parents' perspectives. The use of such a questionnaire is of great value since both reports provide important information from two sources (child vs. parent as proxy) that have already been shown to report different quality of life outcomes (49).

## Conclusions

Our results showed that improving growth of short children by hGH treatment can also affect their short-term HRQOL and might prevent possible psychological consequences of short stature. This information might help to optimize health care in this patient group and to design more individualized care in order to fulfill the needs of patients and parents. Applying the QoLISSY questionnaire in medical practice helped to bring a better understanding of the HRQOL of children and adolescents diagnosed with IGHD, SGA, or ISS.

Although our findings demonstrate that hGH treatment can improve the HRQOL of German children with IGHD or SGA a further follow-up study is warranted to ascertain the long-term effects and the mechanisms through which hGH treatment improves the patients' HRQOL. The elucidation of factors that modify the relationship between hGH therapy and HRQOL are required to adequately determine which patients will derive the most benefit from hGH treatment and to more efficiently allocate health resources and policies.

### Access to the QoLISSY Questionnaire

QoLISSY is a joint initiative between Pfizer Limited and the University Medical Center Hamburg–Eppendorf. Copyright Pfizer Limited all rights reserved. The European QoLISSY instrument, together with comprehensive information of its development and validation process is published in the QoLISSY's User's Manual (31). The Manual, which is available upon request, includes QoLISSY child and parent forms, as well as scoring information (http://www.pfizerpatientreportedoutcomes.com/therapeutic-areas/cv-metabolic/endocrine).

## Data Availability

The raw data supporting the conclusions of this manuscript will be made available by the authors, on request, to any qualified researcher.

## Ethics Statement

Since this study was a multicenter study, the study was approved by the respective ethics committees of the centers, namely by the Hamburg Medical Chamber, the Saxon medical chamber, the Hessen medical chamber, the Nordrhein medical chamber and by the ethics committee of the Friedrich-Alexander University Erlangen–Nürnberg, of the University of cologne, of the University Hospital Magdeburg and of the Ludwig-Maximiliams-University Munich. All subjects gave written informed consent in accordance with the Declaration of Helsinki.

## Author Contributions

JQ, MB, and H-GD contributed conception and design of the study and were main initiators of the study. JQ and JB were responsible for data collection and analysis and wrote the manuscript. NS provided feedback and assistance on the statistical analyses conducted in this study. SW assisted in data collection and in writing of the manuscript. IA, DD, CV, VB, UK, MB, ES, SF-O, AK, and KM were responsible for patient recruitment and data collection. All authors contributed to manuscript revision, read, and approved the submitted version.

### Conflict of Interest Statement

The authors declare that the research was conducted in the absence of any commercial or financial relationships that could be construed as a potential conflict of interest.
